# Effect of induction chemotherapy with cisplatin, fluorouracil, with or without taxane on locoregionally advanced nasopharyngeal carcinoma: a retrospective, propensity score-matched analysis

**DOI:** 10.1186/s40880-018-0283-2

**Published:** 2018-05-10

**Authors:** Guo-Ying Liu, Xing Lv, Yi-Shan Wu, Min-Jie Mao, Yan-Fang Ye, Ya-Hui Yu, Hu Liang, Jing Yang, Liang-Ru Ke, Wen-Ze Qiu, Xin-Jun Huang, Wang-Zhong Li, Xiang Guo, Yan-Qun Xiang, Wei-Xiong Xia

**Affiliations:** 1 State Key Laboratory of Oncology in South China and Collaborative Innovation Center for Cancer Medicine, Sun Yat-Sen University Cancer Center, Guangzhou, Guangdong China; 2 Department of Nasopharyngeal Carcinoma, Sun Yat-Sen University Cancer Center, Guangzhou, Guangdong China; 3 Department of Clinical Laboratory, Sun Yat-Sen University Cancer Center, Guangzhou, China; 4 The Sun Yat-Sen Memorial Hospital, Sun Yat-Sen University, Guangzhou, China; 5 Department of Radiation Oncology, Shanghai Proton and Heavy Ion Center, Shanghai, China

**Keywords:** Nasopharyngeal carcinoma, Induction chemotherapy, Propensity score-matching, Taxane

## Abstract

**Background:**

Available data in the literature comparing different induction chemotherapy (IC) regimens on locoregionally advanced nasopharyngeal carcinoma (NPC) are scarce. The purpose of the present study was to evaluate the outcomes of locoregionally advanced NPC patients who were treated with taxane, cisplatin and 5-fluorouracil (TPF) or cisplatin and 5-fluorouracil (PF) as IC followed by concurrent chemoradiotherapy (CCRT).

**Methods:**

In total, 1879 patients with locoregionally advanced NPC treated with IC and CCRT from a prospectively maintained database were included in the present observational study. We compared overall survival (OS), disease-specific survival (DSS), distant metastasis-free survival (DMFS), and locoregional relapse-free survival, using the propensity score method.

**Results:**

In total, 1256 patients received TPF or PF as IC backbone. The TPF group showed significantly better OS (hazard ratio [HR], 0.660; 95% confidence interval [CI] 0.442–0.986; *P* = 0.042), DSS (HR, 0.624; 95% CI 0.411–0.947; *P *= 0.027) and DMFS (HR, 0.589; 95% CI 0.406–0.855; *P *= 0.005) compared with the PF group in multivariable analyses. Propensity score matching identified 294 patients in each cohort and confirmed that TPF was associated with significantly improved 5-year OS (88.1% vs. 80.7%; *P* = 0.042), DSS (88.5% vs. 80.7%; *P* = 0.021) and DMFS (87.9% vs. 78.6%; *P* = 0.012) rates compared with the PF group. There were no significant differences in locoregional relapse-free survival before or after matching.

**Conclusions:**

In our study, IC with the TPF regimen combined with CCRT showed improved long-term survival for the patients with locoregionally advanced NPC compared with the PF regimen. However, a prospective randomized clinical trial to validate these findings is necessary.

## Introduction

Nasopharyngeal carcinoma (NPC) is a distinct form of head and neck cancer in terms of its etiology, epidemiology, pathology, clinical presentation, and treatment responses [1, 2]. Because of its unique anatomical location and mild, non-specific symptoms, 60–70% of patients present with locoregionally advanced disease at diagnosis [3]. Due to its radiosensitive properties and deep-seated location, radiotherapy is the cornerstone of initial treatment [2, 4, 5]. Moreover, concurrent chemoradiotherapy (CCRT) has been shown to improve survival and is considered the standard-of-care after the landmark intergroup 0099 trial [6]. However, distant metastasis remains a key problem; more than 30–40% of locoregionally advanced NPC patients will develop distant metastasis after standard therapy [7]. Thus, a more efficacious treatment regimen is needed.

Induction chemotherapy (IC) has advantages over adjuvant chemotherapy, including improved tolerability and the early eradication of micrometastases [8, 9]. Recently, several randomized studies and meta-analyses have demonstrated that IC significantly improved disease-free survival (DFS), overall survival (OS) and distant metastasis-free survival (DMFS) [10–14]. Based on these encouraging results, sequential IC followed by CCRT has been included as an option in both the National Comprehensive Cancer Network (category III evidence) [15] and the EHNS–ESMO–ESTRO clinical practice guidelines (category IIB evidence) [16]. Nevertheless, the best IC regimen for NPC has not been defined.

Several randomized trials have demonstrated that OS and progression free survival (PFS) in head and neck cancer were significantly increased by an IC regimen consisting of taxane, cisplatin, and fluorouracil (TPF) compared with cisplatin and fluorouracil (PF) [17, 18]. These trials confirmed TPF as the optimal IC treatment regimen for head and neck cancer. In locoregionally advanced NPC, Sun et al. [11] found that compared with CCRT alone, IC based on TPF plus CCRT significantly improved OS; the 3-year OS rate of the IC group was 92%, which was higher than the group with IC based on PF in previous randomized trials [14, 19]. It remains unknown whether TPF significantly prolongs survival compared to PF as an IC regimen in locoregionally advanced NPC. Thus, we conducted a retrospective, propensity score-matched (PSM) analysis of locoregionally advanced NPC patients who received IC, which either did or did not contain taxane in combination with cisplatin and fluorouracil (TPF or PF, respectively).

## Patients and methods

### Patient selection

We identified patients with newly diagnosed, biopsy proven, stage III–IVb NPC according to the American Joint Committee on Cancer classification system who were treated at Sun Yat-sen University Cancer Center between January 1, 2000 and June 1, 2013. Patients who received IC + CCRT as primary treatment (with either TPF or PF as the IC backbone) were included. Patients who had received other anticancer agents in addition to these initial treatments, had missing medical data, or died during radiotherapy were excluded. Additional information, including demographics, pathological diagnosis, date of diagnosis, imaging results, family history, smoking history, Karnofsky Performance Status (KPS), chemotherapy pattern and drugs, radiation technology and dosage, and follow-up were collected from the hospital information system and paper medical records. T- and N-stages were re-categorized based on the original magnetic resonance imaging (MRI)/computed tomography (CT) imaging, and all patients were re-staged according to the 7th edition of the American Joint Committee on Cancer classification system.

This study was performed in accordance with the Institutional Review Boards of our institution. Written informed consent was obtained from each patient, including signed consent for tissue analysis and consent to be recorded for potential medical research at the time of sample acquisition.

### IC

TPF-treated patients received docetaxel (60 mg/m^2^) or paclitaxel (150 mg/m^2^) and cisplatin (60 mg/m^2^) as a 4-h intravenous infusion on day 1, followed by fluorouracil (600 mg/m^2^) as a 24-h continuous infusion on days 1–5. Patients in the PF arm received intravenous cisplatin (100 mg/m^2^), followed by fluorouracil (1000 mg/m^2^) per day as a continuous 24-h infusion for 5 days. The cycles were repeated every 3 weeks.

### CCRT

RT was given to the nasopharynx and neck using intensity-modulated radiotherapy (IMRT) or two-dimensional radiotherapy (2D-CRT) 5 days/week. The IMRT dose-volume histograms of the treatment targets and critical normal structures were evaluated. The prescribed dose was 70 Gy to the primary tumor, and 60–66 Gy to any involved cervical lymph nodes in 30–32 fractions. 2D-CRT-accumulated radiation doses were 68–76 Gy, with 2 Gy per fraction applied to the primary tumor, and 60–66 Gy applied to involved cervical lymph nodes. Our policy was to accept a plus or minus 5% variation across the target. All patients received a concurrent chemotherapy regimen of cisplatin weekly or every 3 weeks during radiotherapy.

### Follow-up

The date of last follow-up was defined as the last image study and/or clinic visit and/or telephone follow-up. The final follow-up data were updated on October 8, 2015. Patients received follow-up every 3 months for the first 3 years after IC + CCRT, every 6 months for the next 2 years, and then annually, including physical examinations, chest X-ray, abdominal ultrasonography, MRI of the head and neck and/or bone scan, until the end of the study. All survival data were calculated from the date of diagnosis to the date of each event or the last follow-up.

### Statistical analysis

The primary outcomes were OS, DSS, DMFS and locoregional relapse-free survival (LRFS). OS, DSS, DMFS and LRFS were defined as the time from diagnosis to death from any cause, death resulting from NPC or treatment complications, the first distant metastasis, or to the first locoregional relapse. All data, including diagnoses of metastasis and/or local–regional relapse, were audited by the first three co-authors and the last author. Hematological and gastrointestinal reactions were evaluated for acute IC-associated toxicity and were classified based on the National Cancer Institute Common Terminology Criteria for Adverse Events version 4.0.

We analyzed the clinical characteristics and toxicities of the two treatment groups using the Chi squared test. Survival curves for the original unmatched and PSM cohorts were analyzed using the Kaplan–Meier method and log-rank tests. A multivariable Cox regression analysis was used to adjust for IC regimens, sex, age, smoking, the cisplatin dose of concurrent chemotherapy (CDDP dose), time to RT, number of IC cycles, T-stage, N-stage, clinical stage and radiation techniques with a forward logistic regression method and analysis, including covariates that were statistically significant in univariable analysis of the PSM cohort. The results of this analysis are presented as hazard ratios (HRs) with 95% confidence intervals (CIs).

A propensity score analysis was undertaken to adjust for potential biases associated with factors related to receiving specific treatments [20]. Propensity scores were computed by logistic regression for each patient based on the presumed covariates, which included sex, age (≤ 45/> 45), smoking (yes/no), BMI (< 19/19–24/> 24), KPS (< 90/≥ 90), T-stage, N-stage, clinical stage and radiation techniques. The PSM, was generated using all reported covariates with a one-to-one nearest neighbor matching algorithm at a caliper of 0.2. We used SPSS version 22.0 (IBM, Armonk, NY, USA) for statistical analyses, and PSM analyses were performed using R (version 3.2.3). Statistical significance was set at 0.05, and all tests were two-tailed.

## Results

### Study cohort characteristics

**Fig. 1:**
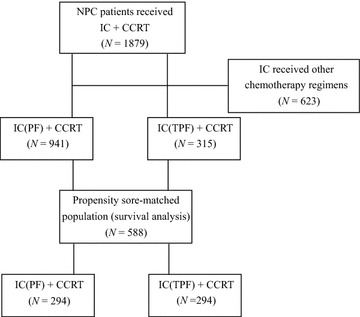
Flow chart of patient selection. *IC* induction chemotherapy, *CCRT* concurrent chemoradiotherapy, *PF* cisplatin and fluorouracil, *TPF* taxane, cisplatin and fluorouracil

Between January 2000 and June 2013, 1879 locoregionally advanced NPC patients were treated with IC + CCRT, among which, 1256 received PF or TPF as the IC backbone (Fig. 1). Among these 1256 patients, 315 (25.1%) were treated with TPF + CCRT and 941 (74.9%) received PF + CCRT. The male:female ratio of the entire cohort was approximately 3:1. Before matching, patients who received TPF as IC were more likely to receive IMRT (*P* < 0.001). After matching, the distribution of radiation techniques was well-balanced between the two groups. Also, all reported parameters were balanced among the two groups, and no statistical differences were detected. Baseline characteristics of the study cohort are shown in Table 1.

**Table 1:** Difference in patient characteristics between the TPF and PF groups in the observational and propensity-matched datasets

Characteristic	Observational dataset [cases (%)]			Propensity score-matched dataset [cases (%)]		
	TPF	PF	*P*	TPF	PF	*P*
Total	315 (25.1)	941 (74.9)		294 (50.0)	294 (50.0)	
Number of DSS events	31 (9.8)	228 (24.2)		28 (9.5)	55 (18.7)	
Gender			0.649			0.114
Male	235 (74.6)	715 (76.0)		224 (76.2)	206 (70.1)	
Female	80 (25.4)	226 (24.0)		70 (23.8)	88 (29.9)	
Age			0.147			0.740
≤ 45	172 (54.6)	558 (59.3)		162 (55.1)	167 (56.8)	
> 45	143 (45.4)	383 (40.7)		132 (44.9)	127 (43.2)	
Histology			0.376			0.362
I	0 (0)	3 (0.3)		0 (0)	2 (0.7)	
II	18 (5.7)	41 (4.4)		18 (6.1)	17 (5.8)	
III	297 (94.3)	897 (95.3)		276 (93.9)	275 (93.5)	
T stage			0.192			0.485
T1	13 (4.1)	22 (2.3)		9 (3.1)	4 (1.4)	
T2	36 (11.4)	126 (13.4)		32 (10.9)	28 (9.5)	
T3	157 (49.9)	436 (46.3)		153 (52.0)	162 (55.1)	
T4	109 (34.6)	357 (38.0)		100 (34.0)	100 (34.0)	
N stage			0.364			0.377
N0	25 (7.9)	103 (10.9)		25 (8.5)	33 (11.2)	
N1	93 (29.5)	276 (29.3)		85 (28.9)	89 (30.3)	
N2	141 (44.8)	420 (44.7)		133 (45.2)	134 (44.6)	
N3	56 (17.8)	142 (15.1)		51 (17.4)	38 (12.9)	
Clinical stage			0.649			0.456
III	165 (52.4)	478 (50.8)		157 (53.4)	167 (56.9)	
IV	150 (47.6)	463 (49.2)		137 (46.6)	127 (43.1)	
KPS			0.837			0.176
70–80	7 (2.2)	25 (2.7)		7 (2.4)	2 (0.7)	
≥ 90	308 (97.8)	916 (97.3)		287 (97.6)	292 (99.3)	
Smoking			0.086			0.144
Yes	119 (37.8)	409 (43.5)		115 (39.1)	97 (33.0)	
No	196 (62.2)	532 (56.5)		179 (60.9)	197 (67.0)	
BMI			0.638			0.602
< 19	33 (10.5)	96 (10.2)		31 (10.6)	29 (9.9)	
19–24	200 (63.5)	574 (61.0)		185 (62.9)	176 (59.8)	
> 24	82 (26.0)	271 (28.8)		78 (26.5)	89 (30.3)	
CDDP dose (mg/m^2^)			0.741			0.438
< 200	188 (59.7)	551 (58.6)		185 (62.9)	195 (66.3)	
≥ 200	127 (40.3)	390 (41.4)		109 (37.1)	99 (33.7)	
Number of IC cycles			0.169			0.729
< 2	54 (17.1)	131 (13.9)		46 (15.7)	42 (14.3)	
≥ 2	261 (82.9)	810 (86.1)		248 (84.3)	252 (85.7)	
Radiation technique			< 0.001			1.0
IMRT	263 (83.5)	262 (27.9)		242 (82.3)	242 (82.3)	
2D-CRT	52 (16.5)	679 (72.1)		52 (17.7)	52 (17.7)	

*TPF* taxane, cisplatin and 5-fluorouracil, *PF* cisplatin and 5-fluorouracil, *DSS* disease-specific survival, *KPS* Karnofsky Performance Status, *BMI* body mass index, *IMRT* intensity-modulated radiotherapy, *2D-CRT* two-dimensional radiotherapy

### Survival outcomes

**Fig. 2:**
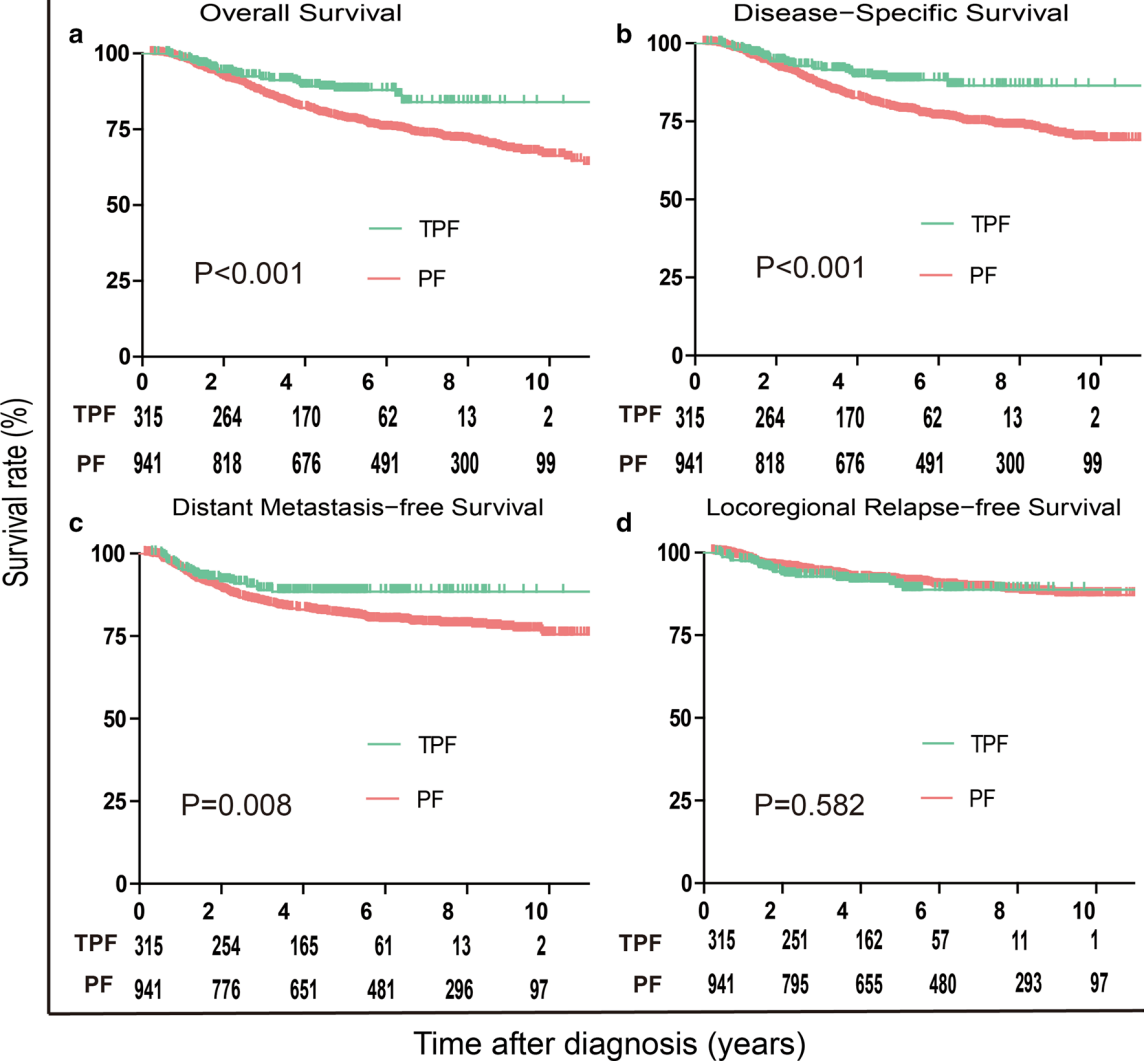
Kaplan–Meier survival curves based on the IC regimens cisplatin and fluorouracil (PF) versus taxane, cisplatin and fluorouracil (TPF) for the entire cohort. **a** Overall survival, **b** disease-free survival, **c** distant metastasis-free survival, and **d** locoregional relapse-free survival. *IC* induction chemotherapy

With a median follow-up of 65 (range: 3–174) months, the 5-year OS, DSS, DMFS and LRFS rates for the entire cohort were 80.1, 80.6, 82.9, and 90.7%, respectively. The 5-year OS, DSS, DMFS and LRFS rates for the TPF vs. the PF group were 87.8% vs. 78% (*P* < 0.001, Fig. 2a), 88.1% vs. 78.7% (*P* < 0.001, Fig. 2b), 88.6% vs. 80.6% (*P* = 0.008, Fig. 2c), and 89.2% vs. 91.0% (*P* = 0.582, Fig. 2d), respectively.

A multivariate analysis was performed using a Cox proportional hazards model to adjust for the various prognostic factors (Table 2). Consistent with the univariate results, multivariate analysis revealed that the TPF-based IC regimen was associated with significantly improved 5-year OS (HR, 0.660; 95% CI 0.442–0.986; *P* = 0.042), DSS (HR, 0.624; 95% CI 0.411–0.947; *P *= 0.027) and DMFS (HR, 0.589; 95% CI 0.406–0.855; *P *= 0.005). There were no significant differences in LRFS (HR, 1.213; 95% CI 0.719–2.047; *P* = 0.469).

**Table 2:** Summary of the multivariable analyses of prognostic factors

Variable	OS		DSS		DMFS		LRFS	
	HR (95% CI)	*P*	HR (95% CI)	*P*	HR (95% CI)	*P*	HR (95% CI)	*P*
IC regimen (PF vs. TPF)	0.660 (0.442–0.986)	0.042	0.624 (0.411–0.947)	0.027	0.589 (0.406–0.855)	0.005	1.213 (0.719–2.047)	0.469
Gender (male vs. female)	0.812 (0.581–1.135)	0.223	0.727 (0.510–1.037)	0.079	0.918 (0.640–1.317)	0.643	0.649 (0.381–1.106)	0.112
Age	1.027 (1.016–1.039)	0.001	1.024 (1.012–1.036)	0.001	1.013 (1.000–1.025)	0.044	1.020 (1.003–1.037)	0.023
Smoking (yes vs. no)	1.041 (0.796–1.363)	0.768	1.003 (0.760–1.323)	0.985	0.944 (0.694–1.285)	0.715	0.976 (0.648–1.472)	0.909
T stage (T1–2 vs. T3–4)	1.393 (1.178–1.648)	0.001	1.231 (0.979–1.549)	0.075	1.306 (1.086–1.570)	0.005	1.420 (1.094–1.842)	0.008
N stage (N0–1 vs. N2–3)	1.308 (1.135–1.509)	0.001	1.177 (1.028–1.349)	0.018	1.547 (1.312–1.824)	0.001	1.146 (0.902–1.456)	0.266
Clinical stage (III vs. IV)	1.185 (0.855–1.642)	0.309	1.630 (1.268–2.097)	0.027	1.012 (0.701–1.460)	0.951	0.909 (0.526–1.572)	0.734
CDDP dose (< 200 mg/m^2^ vs. ≥ 200 mg/m^2^)	0.884 (0.695–1.125)	0.316	0.869 (0.676–1.118)	0.276	1.012 (0.772–1.327)	0.930	1.054 (0.728–1.526)	0.782
Number of IC cycles (< 2 cycles vs. ≥ 2 cycles)	1.089 (0.782–1.518)	0.613	1.121 (0.788–1.593)	0.526	1.217 (0.809–1.829)	0.346	0.899 (0.551–1.467)	0.670
Time to RT (< 60 days vs. ≥ 60 days)	0.874 (0.650–1.173)	0.369	0.840 (0.618–1.142)	0.265	1.102 (0.807–1.506)	0.541	0.857 (0.548–1.339)	0.498
Radiation technique (IMRT vs. 2D-CRT)	1.540 (1.134–2.091)	0.006	1.442 (1.053–1.975)	0.022	1.273 (0.914–1.775)	0.153	1.015 (0.654–1.574)	0.948

*IC* induction chemotherapy, *TPF* taxane, cisplatin and 5-fluorouracil, *PF* cisplatin and 5-fluorouracil, *CDDP dose* the cisplatin dose of concurrent chemotherapy, *RT* radiotherapy, *IMRT* intensity-modulated radiotherapy, *2D-CRT* two-dimensional radiotherapy, *HR* hazard ratio

### Propensity score

**Fig. 3:**
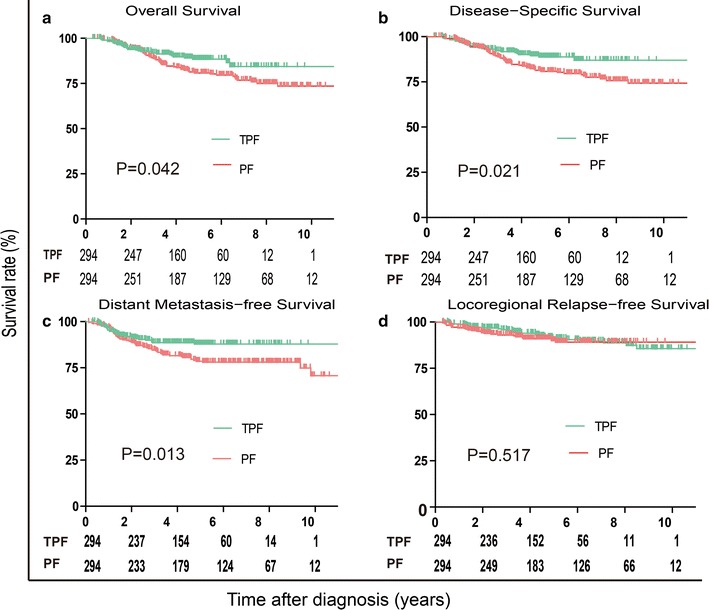
Kaplan–Meier survival curves based on the IC regimens cisplatin and fluorouracil (PF) versus taxane, cisplatin and fluorouracil (TPF) for the propensity-matched cohort. **a** Overall survival, **b** disease-free survival, **c** distant metastasis-free survival, and **d** locoregional relapse-free survival. *IC* induction chemotherapy

Propensity score-matched identified 294 patients in each cohort: the 5-year OS rates for patients treated with TPF and PF were 88.1 and 80.7%, respectively (*P* = 0.042, Fig. 3a), the 5-year DSS rates were 88.5 and 80.7% (*P* = 0.021, Fig. 3b), the 5-year DMFS rates were 87.9 and 78.6% (*P* = 0.012, Fig. 3c), and the 5-year LRFS rates were 89.5 and 91.0% (*P* = 0.517, Fig. 3d). Multivariate analysis was also performed in the PSM cohort to adjust for various prognostic factors (Table 3). This multivariate analysis confirmed that an IC regimen of TPF significantly improved OS (HR, 0.581; 95% CI 0.371–0.910; *P* = 0.018), DSS (HR, 0.543; 95% CI 0.343–0.859; *P *= 0.009) and DMFS (HR, 0.551; 95% CI 0.357–0.850; *P *= 0.007) in the PSM cohort. There were no significant differences in LRFS (HR, 1.179; 95% CI 0.667–2.084; *P* = 0.572).

**Table 3:** Summary of the multivariable analyses of prognostic factors after propensity score matching

Variable	OS		DSS		DMFS		LRFS	
	HR (95% CI)	*P*	HR (95% CI)	*P*	HR (95% CI)	*P*	HR (95% CI)	*P*
IC regimen (PF vs. TPF)	0.581 (0.371–0.910)	0.018	0.543 (0.343–0.859)	0.009	0.551 (0.357–0.850)	0.007	1.179 (0.667–2.084)	0.572
Gender (male vs. female)	1.166 (0.679–2.004)	0.578	1.069 (0.613–1.861)	0.815	1.065 (0.623–1.818)	0.819	0.935 (0.450–1.939)	0.856
Age	1.026 (1.006–1.046)	0.010	1.024 (1.004–1.044)	0.017	1.013 (0.994–1.033)	0.173	1.014 (0.987–1.041)	0.308
Smoking (yes vs. no)	1.020 (0.616–1.687)	0.939	1.004 (0.604–1.667)	0.989	1.118 (0.685–1.823)	0.656	1.061 (0.555–2.028)	0.857
T stage (T1–2 vs. T3–4)	1.349 (0.866–2.101)	0.185	1.345 (0.863–2.096)	0.191	1.449 (1.056–1.987)	0.021	1.434 (0.752–2.733)	0.274
N stage (N0–1 vs. N2–3)	1.285 (0.975–1.693)	0.075	1.319 (0.995–1.748)	0.054	1.652 (1.278–2.134)	0.001	1.125 (0.778–1.627)	0.532
Clinical stage (III vs. IV)	2.193 (1.419–3.390)	0.001	2.277 (1.459–3.552)	0.001	1.137 (0.663–1.042)	0.667	0.891 (0.386–2.057)	0.787
Radiation technique (IMRT vs. 2D-CRT)	1.835 (1.153–2.919)	0.010	1.859 (1.159–2.982)	0.010	2.152 (1.370–3.380)	0.001	1.089 (0.550–2.156)	0.807

*IC* induction chemotherapy, *TPF* taxane, cisplatin and 5-fluorouracil, *PF* cisplatin and 5-fluorouracil, *CDDP dose* the cisplatin dose of concurrent chemotherapy, *IMRT* intensity-modulated radiotherapy, *2D-CRT* two-dimensional radiotherapy, *RT* radiotherapy, *HR* hazard ratio

### Toxicity

Patients toxicities in the PSM cohort were retrospectively evaluated. Regarding hematologic toxicities, the incidences of grade 3/4 anemia (6.5% vs. 2.7%, *P* = 0.047) and grade 3/4 thrombocytopenia (8.8% vs. 3.4%, *P* = 0.009) were higher in the PF group. The incidence of grade 3/4 neutropenia was higher in the TPF group (40.1% vs. 29.6%, *P* = 0.009). With respect to non-hematologic toxicities, grade 3/4 nausea/vomiting occurred more frequently in the PF group (61.2% vs. 48.6%, *P* = 0.003). Neither group experienced serious liver damage or acute renal toxicity. Acute toxicities according to the National Cancer Institute Common Toxicity Criteria, version 4.0 are listed in Table 4.

**Table 4:** Maximum toxicity grade in patients treated with TPF versus PF after matching

Adverse event	PF group [cases (%)]		TPF group [cases (%)]		*P* value
	Grade 0–2	Grade 3–4	Grade 0–2	Grade 3–4	
Neutropenia	207 (70.4)	87 (29.6)	176 (59.9)	118 (40.1)	0.009
Anemia	275 (93.5)	19 (6.5)	286 (97.3)	8 (2.7)	0.047
Thrombocytopenia	268 (91.2)	26 (8.8)	284 (96.6)	10 (3.4)	0.009
Liver injury	291 (99.0)	3 (1.0)	292 (99.3)	2 (0.7)	1.000
Kidney injury	294 (100)	0 (0)	294 (100)	0 (0)	1.000
Nausea/vomiting	114 (38.8)	180 (61.2)	151 (51.4)	143 (48.6)	0.003
Any of the above	109 (37.0)	185 (63.0)	83 (28.2)	211 (71.8)	0.028

*TPF* taxane, cisplatin and 5-fluorouracil, *PF* cisplatin and 5-fluorouracil. Adverse events were graded according to the National Cancer Institute Common Toxicity Criteria, version 4.0

## Discussion

In a large, single institution-based cohort of locoregionally advanced NPC patients who received IC + CCRT, we found that a TPF-based IC regimen significantly improved OS, DSS and DMFS compared with a PF regimen. The TPF regimen also showed acceptable toxicities. To control for potential confounders, a PSM analysis was performed, which also confirmed the consistency of these results.

Updated meta-analyses and systematic reviews of clinical trials have demonstrated a survival advantage to the addition of concomitant chemotherapy to radiotherapy in patients with locoregionally advanced NPC [12]. However, distant metastasis remains a critical issue, as more than 30% of patients with locoregionally advanced NPC develop distant metastases after CCRT, ultimately succumbing to the disease [7, 21]. Benefits have been seen regarding the eradication of distant micrometastases and reduced locoregional failure with IC; the use of chemotherapy as IC before radiation is an attractive model. In a randomized phase II comparison of chemoradiotherapy (cisplatin) with or without IC, Hui et al. [10] reported that the 3-year PFS and OS rates were 88 and 94% in the IC group, respectively, and 60 and 68% in the control group without IC, respectively. Moreover, Sun et al. [11] conducted a randomized phase III study to compare three cycles of induction docetaxel, cisplatin and continuous intravenous fluorouracil followed by CCRT with CCRT alone. IC significantly increased the 3-year failure-free survival, OS and DMFS rates of their patient population. Most recently, a phase III multicenter randomized controlled trial reported that IC improved 3-year DFS (*P *= 0.028) and DMFS rates (*P* = 0.056) compared with CCRT alone in locoregionally advanced NPC [14]. Despite the demonstrated merit of adding IC to CCRT for locoregionally advanced NPC treatment from intense investigations of this approach, studies comparing different IC regimens are scarce.

The PF regimen is the most commonly used IC treatment strategy. In the 1990s, Hareyama et al. [22] conducted a randomized study to compare two cycles of PF-based IC followed by radiotherapy with radiotherapy alone. The 5-year DMFS for patients in the IC + radiotherapy arm was 74% compared with 56% for patients in the radiotherapy-only arm. Moreover, a randomized study of 408 patients designed to compare two cycles of induction floxuridine plus carboplatin followed by radiotherapy with or without concurrent carboplatin (IC + CCRT vs. IC + radiotherapy) for patients with locoregionally advanced NPC was performed, and 5-year OS rates of 70.3 and 71.7% (*P* = 0.734) were found in the IC + CCRT and IC + radiotherapy groups, respectively [19].

Taxanes are microtubule-stabilizing drugs that have been extensively used as effective chemotherapeutic agents for solid tumors treatment [23, 24]. The TAX 323 study was the first to demonstrate the benefits of adding docetaxel to cisplatin and 5-fluorouracil as an IC for locoregionally advanced head and neck cancer. Patients in the TPF group experienced a significant 27% reduction in mortality and an improved median OS of 4.3 months [17]. Later, the TAX 324 study [25] and the GORTEC laryngeal study showed that TPF was significantly better than PF at improving survival, local control, and organ preservation and was associated with manageable toxicity [18, 26].

Because the epidemiology, histology, clinical behavior, and treatment responses of NPC differ from other head and neck cancers, the efficacy of adding a taxane to PF as an IC regimen for locoregionally advanced NPC patients is unclear. In a phase II trial designed to compare TPF with PF as IC regimens in locoregionally advanced NPC patients under the age of 21, Casanova et al. [27] found no differences between the two groups in terms of efficacy or toxicity. This negative result may be attributable to the small sample size and/or the specific adolescent patient population. Recently, a prospective, randomized non-inferiority study evaluated the benefits and side effects of two cycles of TPF as IC compared with two cycles of PF for locoregionally advanced NPC patients. After a short follow-up of approximately 36 months, Jin et al. [28] found no significant differences in PFS between the two groups. However, the small sample size and short-term follow-up period limit these results.

We conducted this retrospective study to compare the outcomes of locoregionally advanced NPC patients who were treated with TPF or PF as IC followed by CCRT in a large cohort with long-term follow-up. We found that TPF-based IC significantly improved long-term OS, DSS and DMFS compared with PF. Interestingly, the 3-year OS and DSS rates for patients treated with TPF and PF were not significantly different. Rather, significant difference presented gradually after 3 years, especially at 5 years, which may partially explain the negative results of the study by Jin et al. In their study, the median follow-up was only 36 months, which may not have been long enough to observe differences between the two groups. Locoregional relapse and/or distant metastasis are the major reasons for treatment failure and death in locoregionally advanced NPC patients [29]. Previous studies have reported that death from locoregional relapse and/or distant metastasis primarily occurs during the first 3 years after diagnosis [11, 14]. In our study, the median OS of patients who developed locoregional relapse or distant metastasis was 38.5 months, and more than 60% of the patients were alive after 3 years of diagnosis. Moreover, in our cohort, more patients died of disease failure after 3 years. Therefore, we believe that greater than 3-year follow-up is needed for NPC patients to determine therapeutic efficacy.

Induction chemotherapy may shrink the primary tumor, providing a wider margin for radiotherapy and reduced organ toxicities. Previous clinical trials have reported that these two IC regimens can achieve satisfactory locoregional control rates [11, 14]. In our study, the 5-year LRFS rates for the whole cohort were both satisfactory and showed no difference between the TPF and PF groups (91.0 and 89.5%, respectively). It is notable that a significantly larger proportion of patients in the PF group underwent 2D-RT techniques. This means, that to some extended, the addition of IC to CCRT was helpful for the local–regional control of NPC regardless of radiation technique. In contrast, the survival benefits of the TPF regimen could also be attributed to a decrease in distant metastasis. Distant metastasis is the most important and lethal outcome for NPC patients; thus, this result suggested that the TPF regimen may provide a better long-term survival and control of distant metastasis in patients who are at a high risk of disease dissemination.

With regard to toxicity, the two regimens were well tolerated, and nearly all patients completed more than two cycles of IC. Acute toxicities during IC were mainly hematologic (neutropenia and anemia), and these incidences were uncomplicated and manageable. Notably, neutropenia was more common in the TPF arm (40.1%) than in the PF arm (29.6%), whereas anemia was more common in the PF arm than the TPF arm. Moreover, the incidence of grade 3/4 nausea/vomiting was higher in the PF group than in the TPF group. The reduced fluorouracil and cisplatin doses administered in the TPF regimen likely minimized gastrointestinal reactions, nut may attributable to the higher anemia rates. Additionally, the additional of a taxane may be responsible for bone marrow suppression.

There were several limitations to this analysis that must be considered. First, although our cohort is likely to be representative of the majority of patients diagnosed with NPC in South China, this was a single-center study. Second, although we used PSM, a method designed to minimize the impact of observed confounders, to account for potential confounders, this was a retrospective analysis, and the results may have been subject to residual confounding variables. For example, we had incomplete data of acute toxicity, such as mucositis, odynophagia, or infectious fever and plasma Epstein–Barr virus DNA, which is one of the major limitations of this study.

## Conclusions

In summary, TPF as an induction regimen before CCRT may improve disease control for locoregionally advanced NPC compared with the PF regimen. A prospective randomized clinical trial to validate these results is necessary.
